# Efficacy and safety of moxidectin and albendazole compared with ivermectin and albendazole coadministration in adolescents infected with *Trichuris trichiura* in Tanzania: an open-label, non-inferiority, randomised, controlled, phase 2/3 trial

**DOI:** 10.1016/S1473-3099(22)00589-8

**Published:** 2023-03

**Authors:** Sophie Welsche, Emmanuel C Mrimi, Jan Hattendorf, Eveline Hürlimann, Said M Ali, Jennifer Keiser

**Affiliations:** a Department of Medical Parasitology and Infection Biology, Swiss Tropical and Public Health Institute, Allschwil, Switzerland; b University of Basel, Basel, Switzerland; c Ifakara Health Institute, Ifakara, Tanzania; d Public Health Laboratory Ivo de Carneri, Chake Chake, Pemba Island, Tanzania

## Abstract

**Background:**

Control efforts against soil-transmitted helminths focus on preventive chemotherapy with albendazole and mebendazole, however these drugs yield unsatisfactory results against *Trichuris trichiura* infections. We aimed to assess the efficacy and safety of moxidectin and albendazole compared with ivermectin and albendazole against *T trichiura* in adolescents living on Pemba Island, Tanzania.

**Methods:**

This open-label, non-inferiority, randomised, controlled, phase 2/3 trial was done in four secondary schools (Kilindi, Kwale, Ndagoni [Chake Chake District], and Kiuyu [Wete District]) on Pemba Island, Tanzania. Adolescents aged 12–19 years who tested positive for *T trichiura* in at least two of four Kato-Katz slides with a mean infection intensity of 48 eggs per gram (EPG) of stool or higher were considered for inclusion. Participants were randomly assigned (21:21:2:2:8) to five treatment groups (8 mg moxidectin and 400 mg albendazole [group 1], 200 μg/kg ivermectin and 400 mg albendazole [group 2], 400 mg albendazole [group 3], 200 μg/kg ivermectin [group 4], or 8 mg moxidectin [group 5]) using a computer-generated randomisation code, stratified by baseline *T trichiura* infection intensity. Study site investigators and participants were not masked to study treatment; however, allocation was concealed to participants. The primary outcome was egg reduction rate (ERR) of *T trichiura* 14–21 days after treatment in the available case population. Moxidectin and albendazole was considered non-inferior to ivermectin and albendazole (control group) when the lower limit of the two-sided 95% CI of the difference was higher than the non-inferiority margin of –2 percentage points. This study is registered with ClinicalTrials.gov, NCT04700423.

**Findings:**

Between March 1 and April 30, 2021, 771 participants were assessed for eligibility. 221 (29%) of 771 participants were ineligible and a further 14 (2%) were excluded. 207 (39%) of 536 participants were randomly assigned to moxidectin and albendazole, 211 (39%) to ivermectin and albendazole, 19 (4%) to albendazole, 19 (4%) to ivermectin, and 80 (15%) to moxidectin. Primary outcome data were available for all 536 participants. The geometric mean ERR of *T trichiura* after 14–21 days was 96·8% (95% CI 95·8 to 97·6) with moxidectin and albendazole and 99·0% (98·7 to 99·3) with ivermectin and albendazole (difference of –2·2 percentage points [–4·2 to –1·4]). No serious adverse events were reported during the study. The most reported adverse events were headache (160 [34%] of 465), abdominal pain (78 [17%]), itching (44 [9%]), and dizziness (26 [6%]).

**Interpretation:**

Our findings show inferiority of moxidectin and albendazole to ivermectin and albendazole against *T trichiura*. However, given the high efficacy, moxidectin coadministration might complement treatment progammes, particularly in areas in which ivermectin is not available

**Funding:**

Bill and Melinda Gates Foundation, reference number OPP1153928.

## Introduction

*Trichuris trichiura* is an intestinal parasite belonging to the soil-transmitted helminths. Trichuriasis is a neglected tropical disease that mostly affects populations in tropical and subtropical climates and is closely linked to poverty.^1^ An estimated 1·5 billion people are infected with soil-transmitted helminths worldwide and infections have resulted in 2 million disability adjusted life-years in 2017.^2^, ^3^ Soil-transmitted helminth infections cause numerous symptoms and morbidity has been linked to infection intensity, chronic infection, and coinfection with several parasites, such as *Plasmodium* spp and *Schistosoma* spp.^4^ Infections can lead to nutrient deficiency, anaemia, and impaired growth, thus children and adolescents are especially clinically vulnerable to these detrimental effects.^5^ Therefore, control programmes often focus on children of preschool (2–4 years) and school age (5–14 years).^6^

**Research in context**

**Evidence before this study**

We searched PubMed and Google Scholar for articles published without language restrictions from database inception to May 6, 2022, using different combinations of the following search terms: “moxidectin”, “albendazole”, “*T trichiura*”, “hookworm”, “soil-transmitted helminths”, and “trial”. Our search identified two clinical trials on the combination of moxidectin and albendazole for the treatment of *Trichuris trichiura* in humans. A dose-finding study that was preceded by a non-inferiority trial found a combination of 8 mg moxidectin and 400 mg albendazole more efficacious than moxidectin alone. However, none of these studies directly compared moxidectin and albendazole with the new recommended combination of ivermectin and albendazole.

**Added value of this study**

We conducted a randomised controlled trial to assess the efficacy of moxidectin and albendazole versus ivermectin and albendazole against *T trichiura* infections in adolescents on Pemba Island, Tanzania. Our study showed superior ERRs and cure rates with a combination of ivermectin and albendazole compared with moxidectin and albendazole. The trial results confirmed that combination chemotherapy has higher efficacy against *T trichiura* than monotherapy and the treatments are well tolerated.

**Implications of all the available evidence**

Ivermectin and albendazole is currently recommended for *T trichiura* and concomitant soil-transmitted helminth infections. Due to an easy administration of moxidectin as a single, weight-independent dose and its benefits over albendazole monotherapy, moxidectin-albendazole combination could serve as a backup in preventive chemotherapy and other control programmes.

The main strategy to eliminate soil-transmitted helminth infections as a public health problem is preventive chemotherapy in the form of mass drug administration programmes that heavily rely on safe, cost-effective, and easily administered drugs.^6^, ^7^, ^8^ The drugs of choice are two benzimidazoles—ie, albendazole and mebendazole—that have shown excellent efficacy against *Ascaris lumbricoides*, but moderate efficacies against hookworm and unsatisfactory results against *T trichiura* infection.^9^, ^10^ Combination chemotherapies gained increasing attention in the past 5 years as a control strategy for soil-transmitted helminth infections to enhance efficacy and slow down drug-resistance development. WHO mentioned the combination of ivermectin and albendazole as a recommended treatment against soil-transmitted helminths in 2017.^11^ A recent multicountry, randomised controlled trial^12^ of ivermectin and albendazole showed superior cure rates compared with albendazole monotherapy in two of three study settings (66% [Laos] and 49% [Pemba Island] for ivermectin and albendazole *vs* 8% [Laos] and 6% [Pemba Island] for albendazole) and a good safety profile with only few minor adverse events reported. Additionally, the combination also improved infection status and intensity 12 months after treatment with intermediate retreatment after 6 months.^13^ Ivermectin is widely used for veterinary deworming and against human lymphatic filariasis and onchocerciasis in control programmes.^14^, ^15^ Since usage across various fields and infections is often associated with an increased risk for the development of resistance, concerns have been voiced on the possible emergence of ivermectin resistance.^16^, ^17^ The ubiquitous use of anthelmintics for livestock and humans increases selection pressure on these parasites. Accordingly, resistance to all major classes of anthelmintics was reported in the veterinary field and remains a threat to human health.^18^, ^19^ Moxidectin, similarly to ivermectin, is a macrocyclic lactone used as an antiparasitic, and was approved by the US Food and Drug Administration in 2018 for the treatment of onchocerciasis in patients older than 12 years.^20^ Evidence from previous trials^21^, ^22^, ^23^ shows that moxidectin might be a good candidate for combination treatment against soil-transmitted helminth infections. Moreover, moxidectin is also active against *Strongyloides stercoralis*; however, non-inferiority has not yet been shown.^24^ Studies comparing moxidectin versus ivermectin against *S stercoralis* are ongoing (NCT04056325 and NCT04848688). Additionally, ivermectin is being considered as a drug used in potential mass drug administration schemes against scabies and the efficacy of moxidectin against the ectoparasite is under investigation.^25^

We aimed to assess the efficacy and safety of moxidectin and albendazole compared with ivermectin and albendazole against *T trichiura* in adolescents living on Pemba Island, Tanzania. To measure long-term effects of moxidectin due to the longer half-life (20–35 days *vs* 18 h for ivermectin), follow-up was conducted at 14–21 days, 5–6 weeks, and 3 months after treatment.^26^, ^27^

## Methods

### Study design and participants

This open-label, non-inferiority, randomised, controlled, phase 2/3 trial was done in four secondary schools (Kilindi, Kwale, Ndagoni [Chake Chake District], and Kiuyu [Wete District]) on Pemba Island, Tanzania. Adolescents aged 12–19 years who tested positive for *T trichiura* in at least two of four Kato-Katz slides with a mean infection intensity of 48 eggs per gram (EPG) of stool or higher were considered for inclusion. On treatment day, adolescents eligible based on stool examination were invited to undergo a clinical examination. Participants presenting with anaemia (lower than 80 g/L haemoglobin), a body temperature higher than 38°C, severe chronic or acute systemic illness (as determined by study physicians), those who received anthelmintic treatment in the past 4 weeks, had a positive pregnancy test, or were planning to become pregnant were not eligible.

The trial was conducted in compliance with the Declaration of Helsinki and Good Clinical Practice guidelines. Ethical approval was obtained from the Zanzibar Health Research Institute (NO.ZAHREC/03/PR/OCT/2020/23) and Ethics Committee Northwest and Central Switzerland (EKNZ, AO_2020–00042). Participants aged 18 years or older provided written informed consent, whereas those younger than 18 years were asked to provide written assent and a guardian or parent provided written informed consent. The trial protocol including the study design and methods were published elsewhere.^28^

### Randomisation and masking

Eligible participants were randomly assigned (21:21:2:2:8) to five treatment groups (moxidectin and albendazole, ivermectin and albendazole, albendazole, ivermectin, or moxidectin) using a computer-generated randomisation code by order of arrival to treatment, resulting in a minimum block size of 54. Two levels of baseline *T trichiura* infection intensity (ie, light at ≤999 EPG and moderate or heavy at ≥1000 EPG) served as a basis for stratification. The trial statistician generating the randomisation sequence was not involved in any field activities. To ensure an allocation close to the anticipated ratio—even when the number of participants in a stratum was low—we developed a randomisation procedure with elements from block randomisation, biased coin design, and covariate constraint randomisation (appendix pp 7–8). Sealed, opaque, and sequentially numbered envelopes containing the treatment group assignment were prepared before the trial start by team members who were not further involved in the trial. Laboratory technicians as primary outcome assessors were masked to treatment allocation. Due to the varying doses and size of tablets, study site investigators and participants were not masked to study treatment. However, allocation was concealed to participants and treatment was handed out individually. Personal data and stool samples were coded with a 4-digit unique identifier for analysis and evaluation.

### Procedures

Participants were asked to provide two stool samples at baseline on two consecutive days, with a maximum 5-day interval. Kato-Katz thick smears using 41·7 mg of stool were prepared in duplicate for each sample and assessed by experienced laboratory technicians under a light microscope. Egg counts of *A lumbricoides*, *T trichiura*, and hookworm were noted and entered using tablets via a data entry mask predefined in CommCare (Dimagi, Cambridge, MA, USA). Approximately 10% Kato-Katz slides were randomly chosen for quality control of *T trichiura* and *A lumbricoides* results. An additional slide was prepared from 10% of samples for immediate quality control of hookworm egg counts.

Participants received either 8 mg moxidectin and 400 mg albendazole (group 1), 200 μg/kg ivermectin and 400 mg albendazole (group 2), 400 mg albendazole (group 3), 200 μg/kg ivermectin (group 4), or 8 mg moxidectin (group 5). Moxidectin (Medicines Development for Global Health, Melbourne, VIC, Australia) was available in tablets of 2 mg with participants receiving 4 tablets each. Ivermectin (Merck Sharp & Dohme, Readington, NJ, USA) was administered in 3 mg tablets as a weight-dependent dose. Albendazole (GlaxoSmithKline, London, UK) was given as a single tablet of 400 mg.

On the treatment day, study physicians performed a physical examination (including a rapid assessment of haemoglobin concentrations to rule out anaemia, measurement of body temperature, and the medical history to assess baseline conditions) of all eligible participants to ensure inclusion criteria were met. Females were tested for pregnancy and asked to confirm that no pregnancy was planned throughout the duration of the study. Study physicians assessed adverse events at 3 h and 24 h after treatment. At the three follow-up timepoints of 14–21 days, 5–6 weeks, and 3 months after treatment, adverse events were assessed retrospectively by trained study team members and considered as possibly related when causality could not be ruled out by other conditions. Severity grading was categorised according to the US National Cancer Institute Common Terminology Criteria for Adverse Events (CTCAE; version 5.0).^29^ When symptoms prevailed at follow-up assessments, participants were taken to a study physician or Chake Chake hospital, provided with the prescribed treatment, and followed up by the study team until symptoms resolved.

A subsample of 60 participants were asked to provide blood samples collected by a finger prick for the assessment of pharmacokinetic parameters between day 0 and 7 after treatment. Pharmacokinetic results will be published elsewhere. Efficacy was assessed 14–21 days after treatment by quadruplicate Kato-Katz from two stool samples collected using the same procedures as for baseline sampling. The same procedures were applied at the other timepoints to determine long-term treatment efficacy. At the end of the study, participants who were still positive for soil-transmitted helminth infections were offered the best available treatment option—ie, ivermectin and albendazole.

### Outcomes

The primary outcome was egg reduction rate (ERR) of *T trichiura* 14–21 days after treatment in the available case population. Secondary outcomes were cure rates (defined as the proportion of participants converted from egg-positive at baseline to egg-negative after treatment) of combination therapy groups compared with monotherapy groups for *T trichiura* 14–21 days after treatment; egg reduction rates and cure rates for *A lumbricoides* and hookworm assessed at 14–21 days, 5–6 weeks, and 3 months after treatment; and tolerability of treatment assessed by type, number, and severity of adverse events. Secondary outcomes were assessed in the available case population.

### Statistical analysis

The primary hypothesis was that moxidectin and albendazole combination treatment is not inferior to ivermectin and albendazole. Simulations using data based on a study by Barda and colleagues^30^ were used to determine the sample size. We estimated that 160 participants would be needed per group to ensure a power of 90% that the upper limit of the two-sided 95% CIs would exclude a difference of more than 2 percentage points (the non-inferiority margin) in favour of the ivermectin and albendazole combination group, assuming a true ERR of 98% in both groups. Accounting for possible loss to follow-up of 10% and an additional safety margin of 20% due to uncertainties in the simulation assumptions, the aim was to enrol 210 participants in each combination treatment group. To test superiority of each combination treatment against respective monotherapies we assumed a cure rate of less than 25% for albendazole or ivermectin monotherapy and less than 40% for moxidectin monotherapy. To detect a statistically significant difference with 85–90% power, 20 participants needed to be enrolled in the albendazole group, 20 in the ivermectin group, and 80 in the moxidectin group. The aim was to enrol 540 participants across all five groups.

The primary non-inferiority analysis was done according to the intention-to-treat principles in the available case population (defined as all participants with any primary endpoint data), with a subsequent per-protocol analysis (defined as all participants with no major protocol deviations, excluding those who did not enter the study because they did not satisfy entry criteria, received no treatment, received the wrong treatment or an incorrect dose, or received concomitant therapy). Eggs per gram of stool were calculated from the geometric mean egg counts multiplied by a factor of 24. For each treatment group, ERRs were calculated using geometric mean egg counts assessed at baseline and 14–21 days after treatment according to the following formula:

$${\text{ERR}}_{\text{geometric mean}}=1-\frac{e^{\frac{1}{\text{n}}\sum \log(\text{EPGfollow}-\text{up}+1)}-1}{e^{\frac{1}{\text{n}}\sum \log(\text{EPGbaseline}+1)}-1}$$

To estimate the difference between ERRs and 95% CIs, a bootstrap resampling method with 5000 replicates was used. The geometric SD was calculated as; e^(SD[log(EPG + 1)])^. Absolute differences between the cure rates were assessed using an exact melded binomial test with mid-p correction, relative differences were estimated using unadjusted regression. Logistic regression with adjustment for baseline infection intensity, age, sex, and weight was also performed. For each follow-up timepoint, ERRs and cure rates were calculated.

Adverse events are presented as frequencies without statistical testing, as recommended by Ioannidis and colleagues.^31^ Data was analysed using R (version 4.0.3) and Stata (version 16). This study is registered with ClinicalTrials.gov, NCT04700423.

### Role of the funding source

The funder of the study had no role in study design, data collection, data analysis, data interpretation, or writing of the report.

## Results

**Figure 1:**
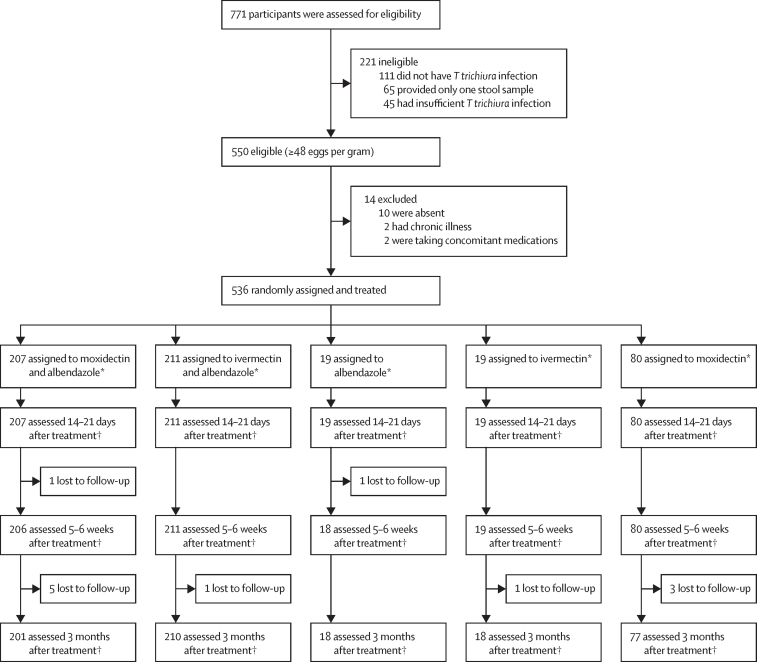
Trial profile Timepoints refer to follow-up. *Assessed for adverse events at 3 h and 24 h after treatment. †Assessed for efficacy and adverse events.

Between March 1 and April 30, 2021, 771 participants were assessed for eligibility (figure 1). 706 (92%) participants provided two stool samples, 550 (71%) were positive for *T trichiura* and had at least 48 EPG infection intensity. 221 (29%) of 771 participants were ineligible and a further 14 (2%) were excluded. Between May 24 and June 3, 2021, 207 (39%) of 536 participants were randomly assigned to moxidectin and albendazole, 211 (39%) to ivermectin and albendazole, 19 (4%) to albendazole, 19 (4%) to ivermectin, and 80 (15%) to moxidectin.

Baseline characteristics are summarised in table 1. The mean age of participants ranged from 15·6 to 15·9 years with a mean of 15·8 years (SD 1·5). 394 (74%) were female and 142 (24%) were male students. Coinfections with other soil-transmitted helminths were common (292 [54%] of 536 were positive for *A lumbricoides* and 173 [32%] had a concomitant hookworm infection). Most *T trichiura* and all hookworm infections were light in intensity, whereas for *A lumbricoides* most participants harboured moderate to heavy infections. More female students were enrolled than males; however, the distribution across treatment groups was balanced.

**Table 1:** Baseline characteristics of trial participants

			**Moxidectin and albendazole (n=207)**	**Ivermectin and albendazole (n=211)**	**Albendazole (n=19)**	**Ivermectin (n=19)**	**Moxidectin (n=80)**
Age, years			15·8 (1·5)	15·8 (1·5)	15·6 (1·2)	15·9 (1·3)	15·8 (1·4)
Sex assigned at birth							
	Females		148 (71%)	163 (77%)	13 (68%)	16 (84%)	54 (68%)
	Males		59 (29%)	48 (23%)	6 (32%)	3 (16%)	26 (33%)
Height, cm			157·1 (7·4)	157·2 (7·3)	159·1 (7·8)	158·9 (7·1)	157·4 (6·8)
Weight, kg			48·3 (8·3)	47·7 (8·2)	50·8 (7·8)	48·1 (6·1)	47·1 (6·5)
*Trichuris trichiura* infection							
	Geometric mean EPG		450 (4)	468 (4)	421 (4)	573 (4)	481 (3)
	Arithmetic mean EPG		975 (1329)	1045 (1509)	1014 (1504)	1670 (3243)	935 (1435)
	Infection intensity*						
		Light	144 (70%)	148 (70%)	14 (74%)	14 (74%)	57 (71%)
		Moderate	63 (30%)	63 (30%)	5 (26%)	4 (21%)	23 (29%)
		Heavy	0	0	0	1 (5·3)	0
Hookworm infection							
	Infected		64 (31%)	70 (33%)	3 (16%)	8 (42%)	28 (35%)
	Geometric mean EPG		125 (4)	112 (4)	128 (8)	94 (5)	89 (3)
	Arithmetic mean EPG		261 (332)	261 (385)	428 (644)	200 (208)	150 (175)
	Infection intensity†						
		Light	64 (100%)	70 (100%)	3 (100%)	8 (100%)	28 (100%)
*Ascaris lumbricoides* infection							
	Infected		106 (51%)	110 (52%)	12 (63%)	14 (74%)	50 (63%)
	Geometric mean EPG		7819 (4)	5934 (5)	8512 (4)	4724 (4)	6765 (4)
	Arithmetic mean EPG		15 823 (18 844)	14 284 (20 566)	17 062 (19 533)	9821 (9162)	11 969 (12 883)
	Infection intensity‡						
		Light	39 (37%)	49 (45%)	4 (33%)	7 (50%)	17 (34%)
		Moderate	61 (58%)	55 (50%)	7 (58%)	7 (50%)	32 (64%)
		Heavy	6 (6%)	6 (6%)	1 (8%)	0	1 (2%)

Data are mean (SD) and n (%). EPG=eggs per gram.

* *T trichiura* infection intensity categorised by mean EPG of stool (light 1–999, moderate 1000–9999, and heavy ≥10000).

† Hookworm infection intensity categorised by mean EPG of stool (light 1–1999, moderate 2000–3999, and heavy ≥4000).

‡ *A lumbricoides* infection intensity categorised by mean EPG (light 1–4999, moderate 5000–49999, and heavy ≥50000).

Primary outcome data were available for all 536 participants (table 2). The geometric mean ERR of *T trichiura* after 14–21 days was 96·8% (95% CI 95·8 to 97·6) with moxidectin and albendazole and 99·0% (98·7 to 99·3) with ivermectin and albendazole. The difference of –2·2 percentage points (–4·2 to –1·4) was large enough to reject non-inferiority and show inferiority of moxidectin and albendazole over ivermectin and albendazole. No major protocol deviations were recorded; therefore, the per protocol population was identical to the available case population.

**Table 2:** ERR and cure rates for *T trichiura*, hookworm, and *A lumbricoides* across different follow-up timepoints

		**Moxidectin and albendazole**	**Ivermectin and albendazole**	**Albendazole**	**Ivermectin**	**Moxidectin**
***Trichuris trichiura* infection**						
Number of participants assessed at 14–21 days		207	211	19	19	80
Geometric mean EPG						
	14–21 days after treatment	14·3	4·6	58·0	33·0	69·7
	Geometric mean ERR	96·8% (95·8 to 97·6)	99·0% (98·7 to 99·3)	86·2% (61·8 to 95·6)	94·2% (89·3 to 97·1)	85·5% (79·7 to 89·8)
Arithmetic mean EPG						
	14–21 days after treatment	94·9	28·5	562·4	68·5	233·1
	Arithmetic mean ERR	90·3% (87·1 to 92·9)	97·3% (96·4 to 98·0)	44·5% (9·6 to 73·2)	95·9% (89·3 to 98·0)	75·1% (67·3 to 81·2)
Cure rates						
	Participants negative at 14–21 days	71	114	5	2	9
	Cure rate	34·3% (27·9 to 41·2)	54·0% (47·1 to 60·9)	26·3% (9·1 to 51·2)	10·5% (1·3 to 33·1)	11·2% (5·3 to 20·3)
Number of participants assessed at 5–6 weeks		206	211	18	19	80
Geometric mean EPG						
	5–6 weeks after treatment	13·5	8·1	104·6	132·3	70·4
	Geometric mean ERR	97·0% (96·0 to 97·8)	98·3% (97·6 to 98·7)	75·7% (40·8 to 90·5)	76·9% (65·6 to 84·7)	85·4% (77·6 to 90·7)
Arithmetic mean EPG						
	5–6 weeks after treatment	100·3	59·4	885·3	252·0	321·5
	Arithmetic mean ERR	89·8% (85·2 to 93·0)	94·3% (92·1 to 96·0)	16·0% (−22·9 to 69·0)	84·9% (63·8 to 90·9)	65·6% (49·4 to 79· 6)
Cure rates						
	Participants negative at 5–6 weeks	77	98	3	0	9
	Cure rate	37·4% (30·8 to 44·4)	46·4% (39·6 to 53·4)	16·7% (3·6 to 41·4)	0% (0·0 to 17·6)	11·2% (5·3 to 20·3)
Number of participants assessed at 3 months		201	210	18	18	77
Geometric mean EPG						
	3 months after treatment	30·8	13·5	93·7	248·3	137·5
	Geometric mean ERR	93·2% (90·8 to 95·0)	97·1% (96·1 to 97·9)	78·2% (55·5 to 90·0)	55·4% (22·3 to 74·2)	71·9% (58·9 to 81·4)
Arithmetic mean EPG						
	3 months after treatment	206·2	102·6	404·7	525·0	533·2
	Arithmetic mean ERR	78·9% (73·0 to 84·2)	90·2% (86·8 to 92·9)	61·6% (38·6 to 76·7)	69·3% (20·2 to 85·3)	44·3% (18·0 to 63·3)
Cure rates						
	Participants negative at 3 months	55	83	2	0	7
	Cure rate	27·4% (21·3 to 34·1)	39·5% (32·9 to 46·5)	11·1% (1·4 to 34·7)	0% (0·0 to 18·5)	9·1% (3·7 to 17·8)
**Hookworm infection**						
Number of participants assessed at 14–21 days		64	70	3	8	28
Geometric mean EPG						
	14–21 days after treatment	1·5	3	0	35·6	16·7
	Geometric mean ERR	98·8% (97·9 to 99·4)	97·4% (95·9 to 98·4)	100%	61·9% (−8·0 to 86·8)	81·2% (58·9 to 91·7)
Arithmetic mean EPG						
	14–21 days	14	26·3	0	108·8	89·6
	Arithmetic mean ERR	94·7% (91·9 to 97·3)	89·9% (84·5 to 94·8)	100·0%	45·5% (−21·8 to 70·0)	40·4% (6·2 to 67·7)
Cure rates						
	Participants negative at 14–21 days	48	44	3	2	9
	Cure rate	75·0% (62·6 to 85·0)	62·9% (50·5 to 74·1)	100% (29·2 to 100·0)	25% (3·2 to 65·1)	32·1% (15·9 to 52·4)
***Ascaris lumbricoides* infection**						
Number of participants assessed at 14–21 days		106	110	12	14	50
Geometric mean EPG						
	14–21 days after treatment	0	0·1	0·4	0	0·2
	Geometric mean ERR	100·0%	100·0%	100·0%	100·0%	100·0%
Arithmetic mean EPG						
	14–21 days after treatment	0	1·9	5	0	104·6
	Arithmetic mean ERR	100·0%	100·0%	100·0% (99·9 to 100·0)	100·0%	99·1% (96·9 to 100·0)
Cure rates						
	Participants negative at 14–21 days	106	106	11	14	49
	Cure rate	100·0% (96·6 to 100)	96·4% (91·0 to 99·0)	91·7% (61·5 to 99·8)	100·0% (76·8 to 100)	98·0% (89·4 to 99·9)

Data are mean (95% CI), unless stated otherwise. ERR=egg reduction rate. EPG=eggs per gram.

A secondary analysis showed that ivermectin and albendazole was superior to moxidectin and albendazole in terms of the secondary outcome cure rate for *T trichiura* 14–21 days after treatment (54·0% *vs* 34·3%, difference of 19·7 percentage points [10·2 to 28·9]; p<0·0001). Estimates from the adjusted logistic regression models were similar to the unadjusted estimates (appendix p 4). The cure rate of moxidectin and albendazole was 8·0 percentage points higher (–15 to 25) than that of albendazole monotherapy; however, the difference was not statistically significant. The cure rate of moxidectin and albendazole was significantly higher than moxidectin monotherapy (difference of 23·0 percentage points [12·6 to 31·8]; p<0·0001). Ivermectin and albendazole resulted in significantly higher cure rates than albendazole monotherapy (difference of 27·7 percentage points [4·0 to 45·1]; p=0·022) and ivermectin monotherapy (difference of 43·5 percentage points [22·4 to 54·8]; p=0·0002). There were no statistical differences in terms of complete response and ERR against *T trichiura* between moxidectin and albendazole and ivermectin and albendazole during 5–6 weeks and 3 months after treatment.

The point estimate cure rates and ERRs for *T trichiura* infection were higher with combination treatments than with either of the three monotherapies across all three follow-up timepoints. Although the difference between cure rates of moxidectin and albendazole versus albendazole monotherapy was not statistically significant at any of the follow-up timepoints, the ERRs of moxidectin and albendazole were significantly higher at all three timepoints for *T trichiura* (table 2).

**Figure 2:**
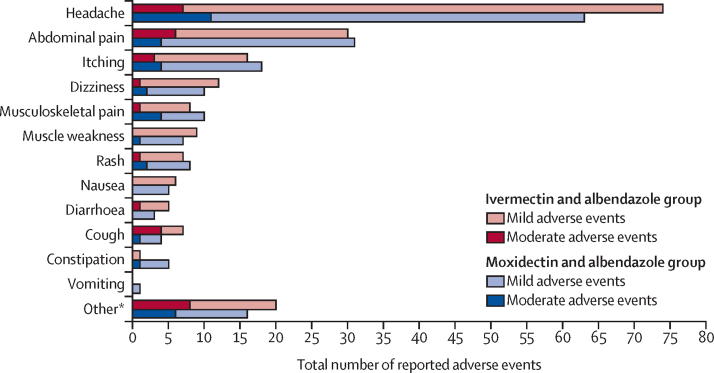
Total number of adverse events Reported in the two combination treatment groups at 3h, 24h, 14–21 days, 5–6 weeks, and 3-month follow-up, stratified by mild or moderate adverse event severity. *Other includes self-reported fever, sleepiness, itching eyes, eye discharge, flu-like symptoms, and ear pain.

No serious adverse events of grade 3–5 were reported in all five treatment groups during the study. The number of participants reporting adverse events are shown in table 3. The type and distribution of adverse events are shown in figure 2 for the two combination treatment groups and in the appendix (pp 5–7) for all treatment groups. Adverse events were predominantly mild (385 [83%] of 465 total adverse events) and a few were moderate (80 [17%]; appendix pp 5–7).

**Table 3:** Number of participants reporting adverse events at each timepoint per treatment group

		**Moxidectin and albendazole**	**Ivermectin and albendazole**	**Albendazole**	**Ivermectin**	**Moxidectin**
Baseline assessment						
	Number assessed	207	211	19	19	80
	Participants with symptoms	7 (3%)	9 (4%)	0	0	2 (3%)
3 h after treatment adverse events assessment						
	Number assessed	207	211	19	19	80
	Participants with adverse events	17 (8%)	25 (12%)	0	2 (11%)	6 (8%)
24 h after treatment adverse events assessment						
	Number assessed	205	211	19	19	80
	Participants with adverse events	36 (18%)	40 (19%)	3 (16%)	2 (11%)	9 (11%)
14–21 days after treatment adverse events assessment						
	Number assessed	207	211	19	19	80
	Participants with adverse events	35 (17%)	41 (19%)	4 (21%)	4 (21%)	13 (16%)
5–6 weeks after treatment adverse events assessment						
	Number assessed	206	211	18	19	80
	Participants with adverse events	20 (10%)	10 (5%)	2 (11%)	2 (11%)	6 (8%)
3 months after treatment adverse events assessment						
	Number assessed	201	210	18	18	77
	Participants with adverse events	10 (5%)	15 (7%)	0	2 (11%)	4 (5%)

Each participant could have more than one adverse event.

Before treatment, 18 (3%) of 536 participants reported symptoms (mainly headache, nausea, or rash). 132 adverse events were reported 24 h after treatment and 149 during the first follow-up after 14–21 days. The most reported adverse events were headache (160 [34%] of 465), abdominal pain (78 [17%]), itching (44 [9%]), and dizziness (26 [6%]). Additionally reported symptoms included itching or watering eyes and various flu-like symptoms, such as throat ache or ear pain (summarised as other; appendix pp 5–7).

## Discussion

Preventive chemotherapy has been a cornerstone strategy in the fight against soil-transmitted helminth infections since the early 1990s. Nevertheless, soil-transmitted helminths remain a major public health problem, partly due to the absence of efficacious, broad-spectrum single-dose treatment options suitable for mass drug administration programmes. Moxidectin is readily available because of repurposing and is one of the few alternatives to albendazole and mebendazole and the combination of ivermectin and albendazole, which are widely used.^12^, ^32^ Moxidectin might be a particularly interesting candidate because the neglected tropical disease community has voiced concerns regarding potential resistance against ivermectin because of its long history of use in mass drug administration programmes against filarial diseases.^18^, ^20^

In this study, we found that moxidectin and albendazole was inferior to ivermectin and albendazole in adolescents infected with T trichiura on Pemba Island, Tanzania. This finding is surprising given our previous results, which showed high efficacy of moxidectin. In more detail, in this study the cure rate of 34·3% for moxidectin and albendazole was considerably lower than those described in two other studies.^23^, ^30^ Barda and colleagues^30^ showed a cure rate of 50·8% and Keller and colleagues^23^ showed a cure rate of 62·5% at the same dose as in our study of 8 mg moxidectin with 400 mg albendazole against *T trichiura* infection. Nevertheless, the ERRs of moxidectin and albendazole observed in our trial are in line with the results of the study by Keller and colleagues,^23^ which was done in the same setting and showed a geometric mean ERR of 97·4%.

Further studies in other settings might reveal useful insights as *T trichiura* might have different drug susceptibility depending on geographical location, which has been suggested in an onchocerciasis study^33^ and observed with albendazole and ivermectin against *T trichiura*.^12^

The cure rate of 26·3% for albendazole was substantially higher in a previous trial conducted on Pemba Island by Hürlimann and colleagues,^12^ who showed a cure rate of 6% (95% CI 4 to 10). This discrepancy in findings might be explained by a smaller sample size of the monotherapy groups in our trial and higher egg counts (100 EPG) used in the study by Hürlimann and colleagues^12^ as an inclusion criterion compared with our study (48 EPG). Nonetheless, the ERRs of moxidectin and albendazole were significantly higher than those of albendazole monotherapy across all three timepoints for *T trichiura*, which together with the consistently higher cure rates point towards an improved treatment effect of the combination.

We had hypothesised that the longer half-life of moxidectin and albendazole compared with ivermectin and albendazole might result in extended effects and hence included a longer efficacy assessment scheme (ie, follow-up at 14–21 days, 5–6 weeks, and 3 months after treatment). No benefit of moxidectin and albendazole compared with ivermectin and albendazole was observed at the timepoints of 5–6 weeks and 3 months after treatment. A potential beneficial effect of the longer half-life of moxidectin was not great enough to outweigh the superiority of ivermectin and albendazole. However, if moxidectin and albendazole were to be applied once or twice a year, as is the case for most mass drug administration schemes against soil-transmitted helminths, the longer half-life of moxidectin might result in a cumulative benefit over time.

This study had some limitations. First, a double-blind study design is favourable to the open-label approach. The reason we did not attempt double-blinding was because of the large number of groups with a highly variable number and shape of tablets in each treatment group. Therefore, we deemed it unethical to expose participants to many various placebos. Furthermore, the laboratory technicians assessing egg counts were fully masked to treatment allocation, which allows for unbiased results. Second, we only included participants aged 12 years or older since moxidectin is currently approved only for this age group, which is a notable limitation of the drug as previous studies have found a high prevalence and infection intensities in children younger than 12 years.^12^, ^32^ Additionally, preschool age children and younger school age children are the main target groups of mass drug administration campaigns and currently could not be reached with the indication of moxidectin. Further studies of moxidectin in children younger than 12 years are ongoing (NCT03962062). Lastly, the small sample size of albendazole monotherapy in our study and large observed 95% CI (9·1 to 51·2) might have inflated the albendazole cure rate and hindered detection of true differences between moxidectin and albendazole versus albendazole alone.

In conclusion, this study showed inferiority of moxidectin and albendazole over ivermectin and albendazole against *T trichiura* contrary to our initial assumption*.* However, the easy, single-dose, weight-independent administration makes moxidectin suitable for large scale soil-transmitted helminth mass drug administration programmes compared with weight-dependent ivermectin administration; hence, further research on the moxidectin and albendazole combination is warranted. Moxidectin might serve as an alternative in areas in which ivermectin is not readily available. To date, ivermectin is not used for mass drug administration schemes targeting soil-transmitted helminths. Moxidectin has yet to be established as a mass-produced drug widely available for preventive chemotherapy programmes; however, in areas where moxidectin can be administered against onchocerciasis, a joint drug request for an integrated neglected tropical disease approach might be worth considering.

## Data sharing

The study protocol is available on ClinicalTrials.gov, NCT04700423. Individual deidentified participant data will be available upon request 1 year after publication. Supporting clinical documents, including approval of the proposal, statistical analysis plan, and informed consent form plan, will be made available upon request immediately after publication for at least 1 year. Access will be granted for researchers who provide a scientifically sound proposal. The sponsor, investigator, and collaborators will approve each proposal based on scientific merit. Requests should be directed to the corresponding author (jennifer.keiser@swisstph.ch). Researchers who request data will need to sign a data access agreement before they are granted access.

## Declaration of interests

We declare no competing interests.
