# Supplementary material for: Efficacy and safety of moxidectin and albendazole compared with ivermectin and albendazole coadministration in adolescents infected with Trichuris trichiura in Tanzania: an open-label, non-inferiority, randomised, controlled, phase 2/3 trial
Source: Lancet Infect Dis. 2023 Mar;23(3):331–40. doi: 10.1016/S1473-3099(22)00589-8 (PMC9946839; doi:10.1016/S1473-3099(22)00589-8)
Supplement: Supplementary appendix [file mmc1.pdf]

THE LANCET
Infectious Diseases

Supplementary appendix

This appendix formed part of the original submission and has been peer reviewed. We post it as supplied by the authors.

Supplement to: Welsche S, Mrimi EC, Hattendorf J, Hürlimann E, Ali SM, Keiser J. Efficacy and safety of moxidectin and albendazole compared with ivermectin and albendazole coadministration in adolescents infected with *Trichuris trichiura* in Tanzania: an open-label, non-inferiority, randomised, controlled, phase 2/3 trial. *Lancet Infect Dis* 2022; published online Oct 28. https://doi.org/10.1016/S1473-3099(22)00589-8.

Contents

Table S1: Egg reduction rates (based on geometric and arithmetic mean) and cure rates against hookworm and *A. lumbricoides* across 5 to 6 weeks and 3 months post-treatment follow-up time-points 1

Table S2: Adjusted logistic regression analysis of moxidectin-albendazole and ivermectin-albendazole combination therapy arms versus albendazole, ivermectin, moxidectin monotherapy arms. 4

Table S3: Total Number of Adverse Events reported among the five treatment arms, stratified by mild or moderate intensity. 5

S1 Text: Random allocation sequence generation for "Efficacy and safety of combination moxidectin and albendazole, ivermectin and albendazole and albendazole alone in adolescents and adults infected with *Trichuris trichiura*: a randomized controlled trial" 8

Table S1: Egg reduction rates (based on geometric and arithmetic mean) and cure rates against hookworm and *A. lumbricoides* across 5 to 6 weeks and 3 months post-treatment follow-up time-points

	Mox-Alb (arm A)	Ivm-Alb (arm B)	Alb (arm C)	Ivm (arm D)	Mox (arm E)
**Hookworm infection**					
**5 to 6 weeks assessment**					
No. of participants assessed at 5 to 6 weeks	64	70	3	8	28
Geometric mean EPG					
5 to 6 weeks after treatment	1.8	3.6	7	39.4	10
Geometric mean ERR (95% CI)	98.6% (97.4-99.3)	96.8% (94.9-98.1)	94.6% (56.9-100)	57.9% (-10.4-84.3)	88.7% (71-95.9)
Arithmetic mean EPG					
5 to 6 weeks after treatment	17.7	33.9	168	116.2	64.5
Arithmetic mean ERR (95% CI)	93.2% (89.4-96.6)	87% (79.7-92.6)	60.7% (56.9-100)	41.7% (-14.9-63.7)	57.1% (20.7-80.5)
Cure rates					
Participants negative at 5 to 6 weeks	46	43	2	2	12
CR - % (95% CI)	71.9% (59.2-82.4)	61.4% (49-72.8)	66.7% (9.4-99.2)	25% (3.2-65.1)	42.9% (24.5-62.8)
**3 months assessment**					
No. of participants assessed at 3 months	62	70	3	8	25
Geometric mean EPG					
3 months after treatment	2.9	5.5	4.8	41.1	20.1
Geometric mean ERR (95% CI)	97.6% (95.9-98.7)	95.1% (91.5-97.3)	96.3% (83.6-100)	56.1% (-31.9-85.5)	76.7% (39.3-92)
Arithmetic mean EPG					
3 months after treatment	26.5	87.6	64	169.5	111.1
Arithmetic mean ERR (95% CI)	89.9% (85.1-94.1)	66.4% (49.6-81.9)	85% (83.6-100)	15% (-72.1-64.5)	26.6% (-38.8-64.5)
Cure rates					

Participants negative at 3 months	41	42	2	2	8
CR - % (95% CI)	66.1% (53-77.7)	60% (47.6-71.5)	66.7% (9.4-99.2)	25% (3.2-65.1)	32% (14.9-53.5)
***A. lumbricoides* infection**					
**5 to 6 weeks assessment**					
No. of participants assessed at 5 to 6 weeks	106	110	12	14	50
Geometric mean EPG					
5 to 6 weeks after treatment	0	0	0	0	0.2
Geometric mean ERR (95% CI)	100%	100%	100%	100%	100%
Arithmetic mean EPG					
5 to 6 weeks after treatment	0	0	0	0	128.8
Arithmetic mean ERR (95% CI)	100%	100%	100%	100%	98.9% (96.2-100)
Cure rates					
Participants negative at 5 to 6 weeks	106	110	12	14	49
CR - % (95% CI)	100%	100%	100%	100%	98% (89.4-99.9)
**3 months assessment**					
No. of participants assessed at 3 months	102	110	12	14	48
Geometric mean EPG					
3 months after treatment	1.1	1	1	0.8	1.6
Geometric mean ERR (95% CI)	100%	100%	100% (99.9-100)	100% (99.9-100)	100% (99.9-100)

Arithmetic mean EPG					
3 months after treatment	95.3	95.3	95.3	95.3	95.3
Arithmetic mean ERR (95% CI)	99.4% (98.9-99.8)	98.6% (97-99.8)	98.4% (95.4-100)	98.1% (93-100)	83.7% (51.4-99.8)
Cure rates					
Participants negative at 3 months	89	97	11	13	42
CR - % (95% CI)	87.3% (79.2-93)	88.2% (80.6-93.6)	91.7% (61.5-99.8)	92.9% (66.1-99.8)	87.5% (74.8-95.3)

Note: Mox-Alb, moxidectin-albendazole; Ivm-Alb, ivermectin-albendazole; Alb, albendazole; Ivm, ivermectin; Mox, moxidectin.

Table S2: Adjusted logistic regression analysis of moxidectin-albendazole and ivermectin-albendazole combination therapy arms versus albendazole, ivermectin, moxidectin monotherapy arms.

	Mox-Alb vs Ivm-Alb				Mox-Alb vs Alb				Mox-Alb vs Mox				Ivm-Alb vs Alb				Ivm-Alb vs Ivm			
	Crude		Adjusted		Crude		Adjusted		Crude		Adjusted		Crude		Adjusted		Crude		Adjusted	
	OR	95% CI	OR	95% CI	OR	95% CI	OR	95% CI	OR	95% CI	OR	95% CI	OR	95% CI	OR	95% CI	OR	95% CI	OR	95% CI
Treatment arm	2.25	1.52;3.35	2.28	1.54;3.4	0.68	0.21;1.87	0.68	0.21;1.87	0.24	0.11;0.49	0.24	0.11;0.48	0.30	0.1;0.83	0.28	0.09;0.76	0.10	0.02;0.36	0.09	0.01;0.34
Age >= 15			1.05	0.69;1.59			0.83	0.47;1.47			0.91	0.53;1.59			1.15	0.64;2.05			1.31	0.72;2.36
Male			0.96	0.61;1.52			0.85	0.45;1.58			1.10	0.61;1.97			1.01	0.54;1.9			1.03	0.54;1.98
Weight [kg]			1.02	0.99;1.04			1.00	0.97;1.04			0.99	0.95;1.02			1.03	0.99;1.07			1.02	0.99;1.06

Note: Mox-Alb, moxidectin-albendazole; Ivm-Alb, ivermectin-albendazole; Alb, albendazole; Ivm, ivermectin; Mox, moxidectin; CI, confidence interval; OR, odds ratio.

Table S3: Total Number of Adverse Events reported among the five treatment arms, stratified by mild or moderate intensity.

**Symptom**	**Baseline**		**3 hours post-treatment**		**24 hours post-treatment**		**14-21 days post-treatment**		**5-6 weeks post-treatment**		**3 months post-treatment**	
**Moxidectin-Albendazole**	**N=207**		**N=207**		**N=205**		**N=207**		**N=206**		**N=201**	
	**mild**	**moderate**	**mild**	**moderate**	**mild**	**moderate**	**mild**	**moderate**	**mild**	**moderate**	**mild**	**moderate**
Headache	2	0	9	1	18	2	11	7	7	1	5	0
Abdominal pain	0	0	6	0	7	0	8	1	5	2	1	1
Itching	0	0	1	0	3	0	5	1	5	2	0	1
Dizziness	0	0	0	0	1	0	3	2	1	0	3	0
Musculoskeletal pain	0	0	1	0	3	2	1	2	0	0	1	0
Muscle weakness	0	0	2	0	3	1	0	0	0	0	1	0
Rash	4	0	0	0	2	0	0	1	0	1	0	0
Nausea	1	0	1	0	2	0	0	0	1	0	0	0
Diarrhea	0	0	1	0	1	0	0	0	1	0	0	0
Cough	0	0	0	0	0	0	0	1	3	0	0	0
Constipation	0	0	0	0	3	0	1	1	0	0	0	0
Vomiting	0	0	0	0	1	0	0	0	0	0	0	0
Other	0	0	0	0	3	0	5	6	2	0	0	0
**Ivermectin-Albendazole**	**N=211**		**N=211**		**N=211**		**N=211**		**N=211**		**N=210**	
	**mild**	**moderate**	**mild**	**moderate**	**mild**	**moderate**	**mild**	**moderate**	**mild**	**moderate**	**mild**	**moderate**
Headache	7	0	16	0	22	0	17	5	3	0	2	2
Abdominal pain	1	0	6	0	9	1	5	5	2	0	1	0
Itching	1	0	3	0	4	1	2	1	2	1	1	0
Dizziness	1	0	1	0	2	0	6	1	0	0	1	0
Musculoskeletal pain	0	0	0	0	6	0	1	1	0	0	0	0
Muscle weakness	0	0	2	0	7	0	0	0	0	0	0	0
Rash	0	0	2	0	1	1	0	0	0	0	3	0
Nausea	2	0	3	0	0	0	0	0	1	0	0	0
Diarrhea	0	0	0	1	4	0	0	0	0	0	0	0
Cough	1	0	0	0	0	0	0	4	2	0	0	0
Constipation	0	0	0	0	1	0	0	0	0	0	0	0
Vomiting	0	0	0	0	0	0	0	0	0	0	0	0

Other	0	0	0	0	0	0	7	6	1	1	4	1
**Albendazole**	**N=19**		**N=19**		**N=19**		**N=19**		**N=18**		**N=18**	
	**mild**	**moderate**	**mild**	**moderate**	**mild**	**moderate**	**mild**	**moderate**	**mild**	**moderate**	**mild**	**moderate**
Headache	0	0	0	0	3	0	2	0	0	0	0	0
Abdominal pain	0	0	0	0	1	0	1	1	0	0	0	0
Itching	0	0	0	0	0	0	0	1	0	0	0	0
Dizziness	0	0	0	0	0	0	0	0	0	0	0	0
Musculoskeletal pain	0	0	0	0	0	0	0	0	0	0	0	0
Muscle weakness	0	0	0	0	0	0	0	0	0	0	0	0
Rash	0	0	0	0	0	0	0	0	0	0	0	0
Nausea	0	0	0	0	0	0	0	0	0	0	0	0
Diarrhea	0	0	0	0	0	0	0	0	0	0	0	0
Cough	0	0	0	0	0	0	0	0	0	0	0	0
Constipation	0	0	0	0	0	0	0	0	0	0	0	0
Vomiting	0	0	0	0	0	0	0	0	0	0	0	0
Other	0	0	0	0	0	0	0	0	2	0	0	0
**Ivermectin**	**N=19**		**N=19**		**N=19**		**N=19**		**N=19**		**N=18**	
	**mild**	**moderate**	**mild**	**moderate**	**mild**	**moderate**	**mild**	**moderate**	**mild**	**moderate**	**mild**	**moderate**
Headache	0	0	1	0	1	0	0	1	0	0	1	0
Abdominal pain	0	0	1	0	0	0	0	1	0	0	0	0
Itching	0	0	0	0	1	0	0	0	1	0	0	0
Dizziness	0	0	0	0	0	0	0	0	1	0	0	0
Musculoskeletal pain	0	0	0	0	1	0	1	0	0	0	0	0
Muscle weakness	0	0	0	0	0	0	0	0	0	0	0	0
Rash	0	0	0	0	1	0	0	0	0	0	1	0
Nausea	0	0	0	0	0	0	0	0	1	0	0	0
Diarrhea	0	0	0	0	0	0	0	0	0	0	0	0
Cough	0	0	0	0	0	0	0	0	0	0	0	0
Constipation	0	0	0	0	0	0	0	0	0	0	0	0
Vomiting	0	0	0	0	0	0	0	0	0	0	0	0
Other	0	0	0	0	0	0	0	2	0	0	1	0

Moxidectin	N=80		N=80		N=80		N=80		N=80		N=77	
	**mild**	**moderate**	**mild**	**moderate**	**mild**	**moderate**	**mild**	**moderate**	**mild**	**moderate**	**mild**	**moderate**
Headache	2	0	4	0	2	0	3	1	2	0	0	0
Abdominal pain	0	0	2	0	2	0	4	0	3	0	0	1
Itching	0	0	0	0	1	0	0	2	1	1	2	0
Dizziness	0	0	1	0	0	0	2	0	0	0	0	0
Musculoskeletal pain	0	0	0	0	3	0	2	0	0	0	0	0
Muscle weakness	0	0	1	0	3	0	0	0	0	0	0	0
Rash	0	0	0	0	0	0	0	0	0	0	0	0
Nausea	0	0	1	0	0	0	0	0	0	0	0	0
Diarrhea	0	0	0	0	1	0	3	0	1	0	0	0
Cough	0	0	0	0	0	0	1	0	0	0	0	0
Constipation	0	0	0	0	0	0	1	0	0	0	0	0
Vomiting	0	0	1	0	1	0	0	0	1	0	0	0
Other	0	0	0	0	0	0	2	1	1	0	1	0

Note: N, Number of participants evaluated; Other, contains self-reported fever, sleepiness, itching eyes, eye discharge, flu-like symptoms and ear pain.

S1 Text: Random allocation sequence generation for "Efficacy and safety of combination moxidectin and albendazole, ivermectin and albendazole and albendazole alone in adolescents and adults infected with *Trichuris trichiura*: a randomized controlled trial"

Author: Jan Hattendorf

The randomization of this randomized trial (anticipated sample size = 540) is characterized by:

i) 2 strata of unknown size (one of them might be small)
ii) An allocation ratio of 21:21:2:2:8 resulting in a minimum block size of 54

A simple block randomization might cause problems because the large block size might result in over- or underrepresented groups if the stratum is small. In the worst case (if the number of participants in a stratum is below 53) that a certain trial arm is not occurring in this stratum at all.

To overcome this limitation, we combined features from block randomization, biased coin design and covariate constraint randomization to ensure that the allocation ratio in each stratum is always close to the anticipated allocation ratio – independent of stratum size.

This is done via the following steps:

1. Define the maximum tolerable difference between the anticipated and actual allocation ratio for each arm
   - We set the maximum allowed difference at 2%, 2%, 6%, 6% and 4%
2. Generate 1'000'000 block randomized sequences of length 540
3. Identify those sequences where the difference between the anticipated and actual allocation ratio is – at any position – never above the maximum allowed difference specified in 1.
   - 74 of the 1 mio sequences satisfied the condition
4. Select randomly 2 from the valid allocation sequences
   - One for each stratum

The R code to generate the allocation sequence is shown below

```
# Function to calculate difference between expected and observed balance
calcDif <- function(x, y, n, obs)
        seq(0,1,length.out=sum(n))-cumsum(y[,obs]==x)/n[x]

# Function for plotting imbalance along the allocation sequence
plotAlloc <- function(y, n, obs = 1, add = F, lty = 1){
  if(add)
        lines(seq(0,1,length.out=sum(n)),cumsum(y[,obs]==1)/n[1],type="l",lty=lty)
  else{
        plot(seq(0,1,length.out=sum(n)), cumsum(y[,obs]==1)/n[1], type="l",lty=lty)
        abline(h=seq(0.1, 0.9, 0.1), v=seq(0.1, 0.9, 0.1), col="gray", lty=3)
        abline(a=0, b=1, col="gray", lty=1, lwd=2)
  }
  for(i in 2:length(n))
        lines(seq(0,1,length.out=sum(n)), cumsum(y[,obs]==i)/n[i],col=i,lty=lty)
}

# Actual seed is kept confidential
set.seed(□□□□□□)

# Anticipated sample size in each trial arm
n <- c(210,210,20,20,80)
sum(n) # 540

# Constraints: define the max allowed imbalance (as fraction) at any position
allowedImbalance <- c(0.02, 0.02, 0.06, 0.06, 0.04)
```

```
# Number of random allocation sequences to generate
loops <- 1000000
# Blocks

block <- unlist(mapply(rep, 1:5, each = c(21,21,2,2,8)))
# Block size
length(block) # 54

# Random sequences. length = 10 x block size
allSeq <- replicate(loops, as.numeric(replicate(10, sample(block))))
dim(allSeq)

# Identify sequences never having an imbalance above the maximum allowed
valid <- rep(NA, loops)
for(i in 1:loops){
 dif <- mapply(calcDif, x=1:5, MoreArgs = list(y=allSeq, n=n, obs=i))
 mabs <- apply(abs(dif), 2, max)
 valid[i] <- all(mabs < allowedImbalance)
}
valid <- which(valid)
length(valid)

# Randomly select 2 of them (1 per stratum)
res <- valid[sample(1:length(valid), 2)]

#plot the sequences
plotAlloc(allSeq, n=n, obs=res[1], lty=1)
plotAlloc(allSeq, n=n, obs=res[2], add=T, lty=2)

# Convert to data frame for export
df <- data.frame(id1=1:sum(n), seq1=allSeq[, res[1]],
                 id2=1:sum(n), seq2=allSeq[, res[2]])
```
